# Effect of Wheat Gluten Films Infused with Mint and Clove Essential Oils on the Shelf Life of Fresh Minced Chicken

**DOI:** 10.3390/foods15020390

**Published:** 2026-01-21

**Authors:** Arsenios Anthomelides, Alexia Gkourogianni, Ioanna S. Kosma, Anastasia V. Badeka

**Affiliations:** Laboratory of Food Chemistry, Department of Chemistry, University of Ioannina, 45110 Ioannina, Greece; arsenisanthom@yahoo.gr (A.A.); alexiagro@yahoo.gr (A.G.)

**Keywords:** wheat gluten, biodegradable film, spearmint, clove, essential oils, antimicrobial agent, antioxidant agent

## Abstract

The need for active biodegradable packaging materials with the ability to improve the microbiological stability of highly perishable foods was investigated in the present study. Specifically, wheat gluten-based films infused with spearmint (*Mentha spicata* L.) and clove (*Syzygium aromaticum* L.) essential oils (EOs)were studied by linking the physicochemical and mechanical properties of the film to the microbiological quality and shelf-life behavior of minced chicken under aerobic refrigerated storage. The packaged samples tested were packaging without film (Control), a wheat gluten film (WGF), WGF with 2% spearmint EO (WGF + 2% SPR), and 2% clove EO (WGF + 2% CL) stored at 4 ± 1 °C for 8 days, under aerobic conditions. Shelf-life extension was evaluated based on established microbiological spoilage criteria, indicating delayed microbial growth in samples packaged with EO-enhanced films compared with the Control. Microbiological analyses (TVC, yeast, *Pseudomonas* spp., *B. Thermosphacta*, *Enterobacteriaceae*, LAB) showed that WGF + 2% CL delayed the time required to reach the spoilage threshold for TVC (7 log CFU/g) by 2 days compared with the Control, while WGF and WGF + 2% SPR extended shelf life by 1 day. Physicochemical properties (pH and objective color) also showed better pH stability and limited color changes in the packaged samples. Mechanical properties resulted in improved film antioxidant activity and flexibility and reduced tensile strength for the EO-enhanced films. Overall, WGFs enhanced with EOs seem to improve minced chicken meat quality during refrigerated storage through the combined effect of antimicrobial activity and modified film properties, highlighting their potential as active packaging materials under the specific conditions studied.

## 1. Introduction

Changes in consumer preferences in recent years, such as the growth of the frozen ready-to-eat market and the increasing demand for minimally processed and preservative-free products, have also led to innovations and developments in the packaging sector [1]. In today’s competitive and constantly evolving market, optimal packaging is a strong advantage for companies to convince consumers to buy a particular brand. Food packaging must perform various functions and meet many requirements and conditions. It must protect food from external environmental conditions such as light, humidity and ambient gases, microbial contamination, extreme temperature changes, insects and rodents, mechanical stress, and dust. Other basic requirements are ensuring adequate nutritional labeling and ease of use for the consumer, e.g., easy opening, resealable lids, and a suitable dosing mechanism. Furthermore, elements related to the marketing and advertising of the product such as a reasonable price, attractive appearance, low environmental impact, suitability for the recycling or refilling of the container and mechanical functionality (e.g., suitability for automatic packaging machines, sealability, etc.) should be considered when designing packaging. However, sometimes, some of the above requirements are contradictory to each other, at least to some extent. The trend towards ensuring food quality and safety without or at least with fewer additives and preservatives demonstrates that packaging plays a particularly important role in this sector: to reduce the incidence of food poisoning and allergies and avoid environmental and economic failure [2].

Recent studies have examined the potential of biodegradable and/or biodegradable materials such as polysaccharide- or protein-based films in combination with antimicrobial compounds to enhance their properties as active food packaging materials. These materials have significant advantages as they are environmentally friendly but also because they can extend the shelf life of food, delay spoilage, and inhibit the growth of microorganisms [3]. Wheat gluten is a large, extended polypeptide polymer without a globular structure and is produced when wheat dough is washed to remove starch granules and water-soluble components. It is a cheap and abundant raw material, and its use as raw material for the development of biodegradable food packaging materials is an interesting approach. As reported in previous studies, wheat gluten has been reported to exhibit film-forming properties capable of forming semi-permeable films to gases and water vapor. Wheat gluten films could thus be applied as food coatings or as edible films in foods naturally containing gluten (e.g., bakery products) to slow mass transfer phenomena, such as water and oxygen, which are known to reduce food quality [4]. Furthermore, in edible films and coatings, substances such as antimicrobial, antifungal, and/or antioxidant agents, colorants, flavors, “beneficial” microorganisms, etc., can be added at the lowest possible concentration to maintain the functionality of the film, mainly on the surface of the food. In this case, the film may act as a carrier that entraps the corresponding substance or microorganism, thus creating conditions for their controlled release [5]. The use of EOs as food additives (flavor enhancers), in perfumery, and in medicine manufacture has wide applications as their antioxidant and antimicrobial activity is exploited. Their application in the food industry is carried out either by directly adding them to food to enhance its properties or by incorporating them into food packaging materials but in concentrations that are organoleptically acceptable by consumers [6,7]. EOs are rich sources of bioactive compounds with potent antioxidant and antimicrobial activity that can be incorporated in active packaging materials to enhance antimicrobial activity against a broad spectrum of microorganisms [8,9,10], and there is increased interest in incorporating EOs into films to improve the shelf life and microbiological safety of foods [11].

Considering the above, the aim of this work was to evaluate the properties of biodegradable films produced from wheat gluten, with and without the addition of EOs (spearmint and clove) while studying the shelf life of minced chicken packaged in these films. It was hypothesized that wheat gluten films enhanced with EOs would exhibit enhanced antioxidant and antimicrobial properties compared with the plain wheat gluten films, resulting in the delayed microbial spoilage of minced chicken samples. Regarding films, a study on their mechanical properties, the objective measurement of color, their antioxidant activity, and their chemical structure by obtaining FTIR spectra was carried out. Finally, a study of physicochemical and microbiological analyses was carried out on the packaged food at predetermined time intervals to estimate its shelf life.

Although wheat gluten-based films incorporating EOs have already been investigated, limited studies have linked the films’ physicochemical and mechanical properties to the microbiological behavior and quality characteristics of minced poultry products during refrigerated storage. Minced chicken was chosen because it is a particularly demanding food matrix, due to its large surface area, disrupted muscle structure, and easy microbial growth, making it an appropriate substrate for evaluating the effectiveness of active packaging systems. Despite wheat gluten films’ advantages (film-forming ability, edibility, biodegradability) and limitations (moisture sensitivity, lack of inherent antimicrobial activity), they exhibit moderate mechanical strength and barrier properties which may be altered, highlighting the need to evaluate their performance in real food systems. Spearmint and clove EOs were selected due to their proven antimicrobial efficacy [12] against spoilage by pathogenic microorganisms commonly associated with poultry, and for their acceptable sensory profiles compared with more intense essential oils.

## 2. Materials and Methods

### 2.1. Chemicals and Reagents

Chemical and reagents used: wheat gluten in powder form (>80% purity, “Ola Bio”, VIOUGEIA Ltd., Agios Stefanos, Attica, Greece), ethanol (Merck kGaA, Darmstadt, Germany), glycerol (Glycerol ≥ 99%, Fisher Chemical, Loughborough, UK), NH_4_(OH) (Ammonia Solution 25%, Merck kGaA, Darmstadt, Germany), methanol (Merck kGaA, Darmstadt, Germany), 2,2-diphenyl-1-picrylhydrazyl/DPPH (TCI Europe N.V, Zwijndrecht, Belgium), buffered peptone water (Biolife, Italiana S.r.l., Milano, Italy), non-selective tryptic glucose yeast agar medium [(TGYA) Biolife, Italiana S.r.l., Milano, Italy], pseudomonas agar base medium (Oxoid, Basingstoke, UK) with the addition of antibiotics cetrimidefucidin–cephaloridine (C.F.C., Oxoid, Basingstoke, UK), streptomycin thallus acetate actidione agar base medium (OXOID, Basingstoke, UK) with the addition of antibiotic (SR0151, OXOID, Basingstoke, UK), violet red bile glucose agar (Biolife, Italiana S.r.l., Milano, Italy), Man–Rogosa–Sharpe agar (MRS, Biolife, Italiana S.r.l., Milano, Italy), chloramphenicol rose bengal agar (RBC, Biolife, Italiana S.r.l., Milano, Italy), hexane (Merck kGaA, Darmstadt, Germany), spearmint EO (>99%, Umakecosmetics, Athens, Greece), and clove EO (>99%, Umakecosmetics, Athens, Greece).

### 2.2. Preparation of Wheat Gluten Films

Three types of films were prepared: wheat gluten films (WGF) and wheat gluten films with the addition of 2% EOs spearmint (WGF + 2% SPR) and clove (WGF + 2% CL). The conditions described below are the result of preliminary tests to optimize their production conditions. In detail, 15 g of wheat gluten in powder form was dissolved in 72 mL of ethanol and stirred for 10 min. Then 5 mL of glycerol acting as a plasticizer was added and stirring continued at ambient temperature until the solution was completely homogenized. The solution was then heated under continuous stirring in a magnetic stirrer (AREX Heating Magnetic Stirrer, VELP SCIENTIFICA, Usmate Velate, MB, Italy) followed by addition initially of 48 mL d. H_2_O and then of 12 mL NH_4_(OH) to adjust the pH to 10.50 to denature the gluten proteins. Heating under stirring continued until the final temperature of the solution reached 75 °C ± 1 °C. Adjustment of pH to 10.5 and temperature of solution heating to 75 °C was conducted in order to promote protein unfolding and enhance film-forming ability, as previously reported for wheat gluten systems [13,14].

In the case of EO-enhanced films, after adjusting the pH of the solution, an appropriate amount of the corresponding EO (spearmint or clove) was added to a final concentration of 2% *v*/*v* relative to the total film-forming solution. A concentration of 2% *v*/*v* was selected based on preliminary tests and literature research balancing antimicrobial efficacy, sensory acceptability, and film integrity [15,16]. Dose–response effects were not examined and represent a limitation of this study. After EO addition, the film-forming solutions were stirred continuously in order to promote uniform dispersion before casting. An appropriate amount of the above solution was plated on a plastic circular dish (diameter of 14.7 cm) and then placed in a chamber (WTB BINDER, Labortechnik GmbH, Tuttlingen, Germany) at 32 °C for 48–72 h for drying. The prepared films (Figure S1) were put on filter papers and stored at 25 °C for 48 h for further use.

### 2.3. Film Characterization

#### 2.3.1. Thickness Measurement of the Prepared Films

Film thickness was measured using a portable digital micrometer (Electronic Outside Micrometer, IS 13109 INSIZE Co., Ltd., Suzhou, China). Thickness was measured at multiple random locations to account for local variability, following common practice in film characterization. Films were measured at 5 different, random points while 3 different films of each type were used for data collection. The mean and standard deviation (STD) were calculated.

#### 2.3.2. Measurement of Film Color

Film color was measured using a Hunter Lab colorimeter (model DP-9000, D25 L Optical Sensor Colorimeter, Reston, VA, USA) and values were obtained for the parameters L* (lightness), a* (redness), and b* (paleness). Circular films from each type were placed on glass plates and four measurements were collected by rotating the plate by 90°. The measurements were triplicated, and the mean and STD were calculated.

#### 2.3.3. Determination of Films’ Antioxidant Activity

For the determination of films’ antioxidant activity, 50 mg of each film was mixed with 10 mL of methanol, stirred with a magnetic stirrer for 1 h, and incubated at room temperature for another 1 h. Centrifugation was then performed for 10 min, at 4000 rpm, to separate the insoluble solids and 1 mL of this solution was added to 4 mL of DPPH solution (0.1 mM in methanol), mixed, and the final solution was stored in the dark for 30 min. After 30 min, the absorbance was measured at 517 nm against a methanol blank using a spectrophotometer (Infinite M Nano, Tecan GmbH, Grödig, Austria). The following formula was used to determine the antioxidant activity of the films:

$$\%AA=\frac{Ao-As}{Ao}*100$$

where Ao is the absorbance of the DPPH solution and As is the absorbance of the film solution. The measurements were triplicated, and the mean and STD were calculated.

#### 2.3.4. Measurement of Films’ Mechanical Properties

For films’ mechanical properties measurements, at least 10 samples (1.5 cm × 10 cm) of each type were analyzed using an Instron instrument (Model 4411, Instron, High Wycombe, UK). The measurements were carried out according to the D882 method of the American Society for Testing and Materials (ASTM) [17], at ambient temperature, with a crosshead speed of 500 mm/min and a vice distance of 5 cm. From the tensile measurements and the stress–strain diagrams, the values for the following parameters were obtained: % elongation at break (strain), tensile strength at break, and Young’s modulus (modulus of elasticity). The mean and STD were calculated.

#### 2.3.5. FT-IR Analysis

Attenuated Total Reflectance–Fourier Transform Infrared (ATR-FTIR) spectroscopy was used to analyze the films’ chemical structure, using a Cary 630 model (Agilent, Santa Clara, CA, USA). Each film was subjected to 16 scans at 4 cm^−1^ from 4000 to 400 cm^−1^ at ambient temperature.

### 2.4. Sample Preparation and Packaging

Fresh minced chicken, supplied by the local industry “PINDOS S.A.” located in Ioannina, Greece was immediately transported to the Food Chemistry Laboratory of the University of Ioannina under refrigeration in an isothermal bag. Subsequently, portions of 90 g of minced chicken were formed in the shape of a burger following all sanitary protocols. Four types of samples were prepared: chicken burger without film (Control), chicken burger covered by 2 WGF (top and bottom side), and, similarly, chicken burger covered by 2 WGF + 2% SPR and chicken burger covered by 2 WGF + 2% CL. After being coated with the prepared films, all samples were placed in polystyrene trays and wrapped with a transparent low-density polyethylene (LDPE) film (Figures S2 and S3) and the observed differences were attributed to the presence and composition of WG films. The LDPE overwrap was used in order to prevent moisture loss and external contamination. Finally, the samples were stored at 4 °C ± 1 °C for 8 days. Oxygen concentration in the package headspace was not monitored, representing a limitation of this study. Sampling was carried out as follows: 0, 2, 4, 6, 8 (for each type of packaging, 4 identical samples were prepared, 1 for each sampling day). Sample preparation and handling were conducted under aseptic conditions in order to minimize external contamination. The experiment was replicated 2 times.

### 2.5. Microbiological Analyses

Microbiological analyses of the packaged samples were carried out according to the official methods of analysis of the American Public Health Association [18]. Microbiological analyses were performed under aseptic conditions following standardized dilution and plating procedures. Specifically, for the determination of microbial counts, 25 g of minced chicken samples was aseptically transferred into individual Stomacher bags (Seward Medical, Worthing, UK) containing 225 mL of buffered peptone water (BPW, 0.1%) and homogenized for 60 s using a Lab Blender 400, Stomacher (Seward Medical, Worthing, UK), at room temperature. For each sample, further serial decimal dilutions were prepared in BPW solution (0.1%). Then 0.1 mL of these serial dilutions of minced chicken homogenates was spread onto the surface of agar plates. The following groups or microorganisms were studied: total viable counts (TVCs), yeasts, *Brochothrix thermosphacta*, *Pseudomonas* spp., *Enterobacteriaceae*, and Lactic Acid Bacteria (LAB).

### 2.6. Physicochemical Analyses

pH and objective color parameters were measured according to the methods described by Tsironi et al. [19]. Minced chicken samples (20 g) were completely homogenized with 10 mL of distilled water, and immersion of the electrode and determination of pH followed using a pH meter model HD 3456.2 (DeltaOHMSrl, Selvazzano Dentro, Italy).

Color parameters were measured to assess the color changes during the shelf life of the samples using a Hunter Lab colorimeter model DP-9000 (Reston, VA, USA). Approximately 70 g of minced chicken sample was placed on a glass plate and the parameters L* (brightness), a* (redness), and b* (yellowness) were measured. For each value, the plate was rotated approximately 60° to determine the color on all sides of the meat mass.

### 2.7. Volatile Compounds of the Essential Oils

Identification of volatile compounds of clove and spearmint EOs was conducted according to the method described by Karakosta et al. [20]. Specifically, a 0.1% solution of each EO was prepared in hexane and analyzed using an Agilent 7890A series gas chromatograph equipped with an Agilent 5975C inert XL MSD mass selective detector (Wilmington, DE, USA). The column was DB5-MS (60 m × 320 µm × 1.0 µm), temperature program: 35 °C/3 min, 5 °C/min to 250 °C for 3 min. Helium was the carrier gas, flow rate: 1 mL/min., 30:1 split ratio. The identification of the volatile compounds was made by comparing their mass spectra to those of the Wiley Library.

### 2.8. Statistical Analysis

Experiments were replicated twice (biological replicates consisted of independently prepared samples) while analyses were run in triplicate (corresponding to repeated measurements of the same sample) for each sampling day per treatment. All analyses data were expressed as mean values ± standard deviations and subjected to analysis of variance (ANOVA) with Tukey’s multiple range tests using the MINITAB software package version 18.0 [21].

## 3. Results and Discussion

### 3.1. Film Characterization

#### 3.1.1. Film Thickness

Film thickness, expressed in micrometers, fell within the acceptable limits for a film intended for food packaging (10–300 μm). As shown in Table 1, the WGF and the WGF + 2% SPR have similar thicknesses, while the WGF + 2% CL shows a statistically significant increase (*p* < 0.05) which may be due to the addition of EO during the homogenization process or attributed to formulation effects. A possible filling of the voids within the gluten protein network perhaps increased the thickness to some extent. However, this increase is not high and, in any case, did not exceed the maximum desired limit of 300 μm.

As recorded by Khashayary et al. [22], the thickness of samples prepared from wheat gluten film with the addition of various antimicrobial agents such as vanillin, salicylic acid, and others was found to be similar to those of the present study and ranged between 180 and 290 μm. On the other hand, Rivera et al. [23], who studied wheat gluten films infused with turmeric and cinnamon EOS, found lower values of film thickness (91–111 μm), with the thinnest film being the WFG, while the thickness increased with the addition of the EOS.

#### 3.1.2. Film Color

L*, a*, and b* values for the respective parameters of the prepared wheat gluten films are presented in Table 2. Between WGF and WGF + 2% SPR, there were no significant differences (*p* > 0.05), while WGF + 2% CL showed differences for all three color characteristics. Specifically, the value regarding the L* parameter for the WGF + 2% CL film was almost 3 units lower than that of the other two samples, implying that the WGF + 2% CL films were darker. This may be due to the color of the clove EO added during their preparation, while the a* parameter was at almost similar values, without changing significantly the red hue, and b* parameter ranges at higher values, giving a more yellow hue. These differences may be due to the origin of the raw material, i.e., wheat gluten, which also affects the final color characteristics, or pH adjustments.

Films prepared from wheat gluten by Duan et al. [24] showed higher L* and a* parameters (94.90 ± 0.04, and −1.87 ± 0.05, respectively) while b* recorded lower values (6.42 ± 0.15) compared with the present study. Similarly, Micard et al. [25], who studied the properties of physically and chemically treated wheat gluten films, found higher values for the L* and a* parameters while b* was slightly lower in the simple films.

It is worth mentioning that the incorporation of EOs was carried out under continuous stirring before casting in order to avoid a heterogeneous distribution within the gluten matrix. The absence of phase separation and consistent mechanical behavior between replicates suggests a relatively homogeneous distribution. However, possible microscale heterogeneities cannot be excluded and may partly explain the variability in thickness and color values as well as antimicrobial efficacy.

#### 3.1.3. Film Antioxidant Activity

It is evident that the addition of the EOs significantly increases the antioxidant activity of the films compared with WGF due to the chemical compounds with strong antioxidant and antimicrobial activity which are naturally present in EOs. The results of the present study (Table 3) showed a statistically significant difference (*p* < 0.05) between the two films fortified with EOs (WGF + 2% SPR recorded 50.22 ± 11.47%, and WGF + 2% CL 88.76 ± 1.19%). These differences reflect the distinct composition and concentration of bioactive compounds in clove vs. spearmint EOs.

Similar results to those of the present study were reported by Haniye et al. [26] who developed and characterized antimicrobial and antioxidant films based on wheat gluten, which were infused with free and encapsulated *Origanum majorana* L. EO. Wheat gluten films which had a similar value to that of the present study exhibited the lowest antioxidant activity compared with the others (36.02%). The addition of *O. majorana* EO in free and encapsulated forms increased the antioxidant activity of the films, with the films containing free EO exhibiting the highest antioxidant activity (46.69 ± 0.80%) compared with the encapsulated form (43.43 ± 0.86%).

#### 3.1.4. Mechanical Properties

Films intended for flexible food packaging types should exhibit satisfactory strength. An important parameter for films’ resistance to deformation is the % elongation or % elongation at break (strain). The breaking point is defined as the moment just before the material collapses. Another important parameter is the stress at the breaking point, or tensile strength, expressed in MPa. As can be seen, this parameter expresses the strength exerted on the test piece until it collapses. Finally, Young’s modulus or elasticity coefficient is expressed in MPa and describes a very important physical property of plastic films, namely stiffness/elasticity. Higher values of Young’s modulus imply a higher stiffness of the material. Table 4 shows the values for the three parameters relating to the mechanical properties of the films produced.

Regarding elongation at break, it is observed that the values for WGF and WGF + 2% SPR films are similar, while the value for WGF + 2% CL film is significantly higher. It is evident that the addition of clove EO enhances the elasticity of the film, as the higher the percentage of strain, the more the material can be elongated. The addition of clove oil acts as a plasticizer, imparting elasticity to it. As far as tensile strength is concerned, it appears that the highest value (432.0 ± 47.6 MPa) is recorded for the WGF, with a relative difference from the other two. A higher tensile strength value implies greater material strength. The addition of EOs negatively affects the strength of the films by reducing it, probably because they increase its elasticity and, as a result, reduce the ability to withstand a large load before collapsing. In the case of Young’s modulus, a high value (4107.5 ± 205.8 MPa) is observed in the WGF, followed by WGF + 2% CL (3165.5 ± 37.5 MPa), while the lowest value of the three types was for the WGF + 2% SPR (2786.7 ± 187.0 MPa). A higher Young’s modulus value implies greater material stiffness. Thus, it appears that the most rigid film is WGF. The EO-enhanced films are more flexible, which is likely due to the nature of the oils acting as plasticizers, thereby increasing chain mobility and elongation, making the protein structure of the films more flexible, while simultaneously disrupting the protein network, leading to reduced tensile strength. The increased elasticity due to the incorporation of EOs may improve the conformity around the minced chicken sample, potentially enhancing the barrier effects under aerobic storage conditions.

The results recorded in the present study agree with those recorded by Magied et al. [27] who used prepared films and gluten coatings to package frozen strawberries (*Fragaria ananassa*) and found that films with the same or similar percentages of glycerol as in the present study had strain values of 249.37% (40% glycerol) and 253.40% (50% glycerol), which are quite similar with the mean value for the WGF of the present study (244.17 ± 21.42%). On the other hand, Mojumdar et al. [28], who studied edible films made from wheat gluten protein (WGP) in terms of both thermal and mechanical properties, recorded higher strain values (310–331%), with the reported values in the literature data for protein films ranging from 1 to 260%.

#### 3.1.5. FT-IR Analysis

Figure S4 shows the three different spectra obtained from the three types of films, WGF (blue line), WGF + 2% SPR (yellow line), and WGF + 2% CL (orange line). The spectra of all films largely overlap across the entire wavenumber range (400–4000 cm^−1^), without the appearance of new absorption bands or significant peak shifts after the incorporation of EOs. The absence of new characteristic peaks suggests that the addition of EOs did not lead to the formation of new covalent chemical bonds within the WG matrix. This observation suggests that EOs were mainly physically entrapped within the protein network rather than chemically interacting with gluten components. Possible non-covalent interactions, such as hydrogen bonding or van der Waals forces, cannot be excluded but were not detected by FTIR analysis. The main absorption bands were observed at 3500–3000 cm^−1^ as a broad peak, corresponding to O–H stretching vibrations of alcohol groups and asymmetric and symmetric N–H stretching vibrations of amino groups. Additional peaks at approximately 2940 cm^−1^ and 2880 cm^−1^ were attributed to C–H stretching vibrations of alkanes. The characteristic absorption bands of wheat gluten proteins, associated with amide I, II, and III vibrations, remained intact, suggesting that EOs were physically entrapped rather than chemically bonded, allowing potential controlled release during storage. Specifically, amide I, II, and III vibrations were detected at approximately 1620 cm^−1^, 1540 cm^−1^, and 1450 cm^−1^, respectively. In the fingerprint region, the moderate absorption at 1100 cm^−1^ and the strong absorption at 1040 cm^−1^ were assigned to C–O stretching vibrations of C–O–H and C–O–C groups of the glucose ring. The low-intensity peak observed at approximately 921 cm^−1^ was related to Si–O–Si bonds, consistent with previous reports (Chen et al. [29], Khashayary et al. [22]).

### 3.2. Microbiological Analyses

The microbiological analysis determined the initial microflora of fresh minced chicken stored at 4 ± 1 °C, consisting of yeasts, *Br. thermosphacta*, *Pseudomonas* spp., *Enterobacteriaceae,* and Lactic Acid Bacteria (LAB). The dynamics of these microorganisms as well as their contribution to the final microflora was influenced by various factors, including the type of packaging used. As storage was carried out under aerobic conditions, the direct contact of the films with the meat surface likely modified the local oxygen availability. Wheat gluten films, as mentioned above, exhibit moderate oxygen barrier properties, potentially affecting the dynamics of microbial growth.

#### 3.2.1. Total Viable Counts (TVCs)

Shelf life was defined based on microbiological thresholds commonly applied to minced poultry, particularly TVC values exceeding 7 log CFU/g. [30]. Under these criteria, EO-enhanced films extended shelf life by approximately 2 days compared with the Control. Although modest, this extension is considered significant for minced poultry products, which are characterized by a short shelf life. Even a limited shelf-life extension can reduce food waste, enhance distribution flexibility, and improve safety margins in retail settings.

**Figure 1:**
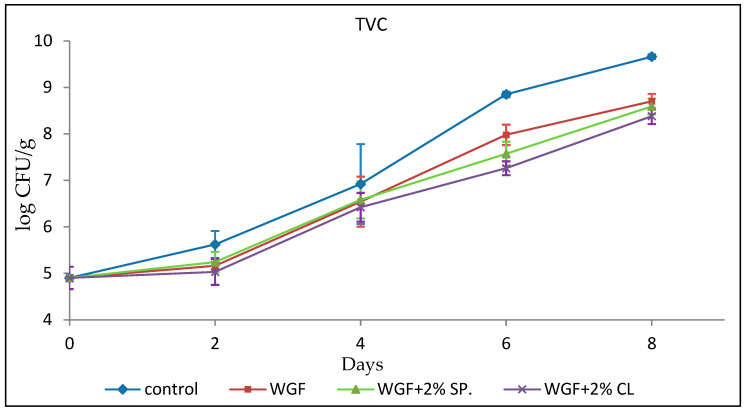
Growth of TVCs in minced chicken packaged in different types of films during storage.

The initial population of TVCs (Figure 1) in minced chicken, i.e., the population on the day the sample was transported from the factory to the laboratory (day 0), was 4.90 ± 0.24 log CFU/g. This observation is consistent with the work of Gertzou et al. [31] and Chouliara et al. [32], who reported that the initial population of TVCs in fresh chicken was found to be 4.65 log CFU/g and 4.28 log CFU/g, respectively. As expected, the microbial load of all samples increased over time.

TVCs reached the limit of 7 log CFU/g on the fourth day for the sample that was not covered with any film (Control), approximately on the fifth day for WGF and WGF + 2% SPR film, while for the sample coated with wheat gluten film enhanced with 2% *v*/*v* clove EO (WGF + 2% CL) the limit of 7 log CFU/g was exceeded on the sixth sampling day. This demonstrates the contribution of biodegradable wheat gluten films to inhibiting the growth of microorganisms that dominate food and thus to extending shelf life. Another finding is that on the sixth day, the number of bacteria begins to decrease in the samples coated with EO-enhanced films (WGF + 2% SPR and WGF + 2% CL) compared with the WGF-coated sample, indicating the antimicrobial effect of EOs which prevent bacteria growth to some extent.

These results are consistent with those of Raeisi et al. [33], who studied the effect of sodium alginate coating films incorporating antimicrobial agents (nisin) and EOs (cinnamon and rosemary) on the microbial quality of chicken. TVC values of all samples increased during storage at 4 °C. The sample that was not covered with any film and the one covered with sodium alginate had the highest rate of microbial growth during storage, while the lowest rate was observed in sodium alginate film packaging with the simultaneous addition of cinnamon and rosemary essential oils (CEO + REO), rosemary and nisin (REO + N), and cinnamon and nisin (CEO + N). Other literature data are in accordance with the results of the present study [29,34] regarding edible wheat gluten films incorporated with EOs and antimicrobial agents.

#### 3.2.2. Yeasts

**Figure 2:**
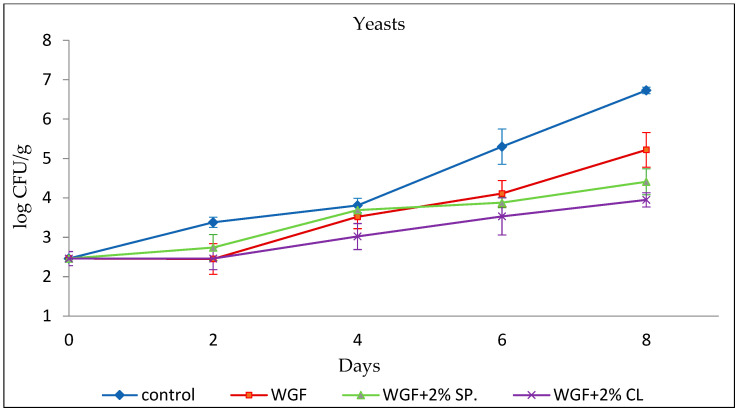
Growth of yeasts in minced chicken packaged in different types of films during storage.

Yeasts belong to a category of microorganisms that may cause spoilage mainly on the surface of chicken stored under aerobic conditions [35]. The initial population of yeast in minced chicken was 2.46 ± 0.18 log CFU/g, which is considered relatively low (Figure 2). As in the case of TVCs, within days, an increase in this microorganism is observed in all types of packaging, with the only exception being on the second day, when the values are similar to those on day 0. On the eighth day of the experiment, the yeast populations for the Control, WGF, WGF + 2% SPR, and WGF + 2% CL samples were 6.73 ± 0.08, 5.22 ± 0.44, 4.41 ± 0.33, and 3.95 ± 0.18 log CFU/g, respectively.

These results are in agreement with Raeisi et al. [33] who examined the effect of sodium alginate coating membranes incorporated with antimicrobial agents and EOs (nisin, cinnamon, and rosemary) on the microbial quality of fresh chicken. Yeasts values increased during storage, reaching a maximum of 6.7 and 6.5 log CFU/g for the sample without any coating and for the one coated by a sodium alginate film, respectively. Samples packaged in films enhanced with a combination of essential oils such as cinnamon and nisin, rosemary and nisin, cinnamon and rosemary, recorded values of 5.1, 5.5, and 5.9 log CFU/g, respectively, which were significantly lower than the previous two treatments.

#### 3.2.3. *Brochothrix Thermosphacta*

**Figure 3:**
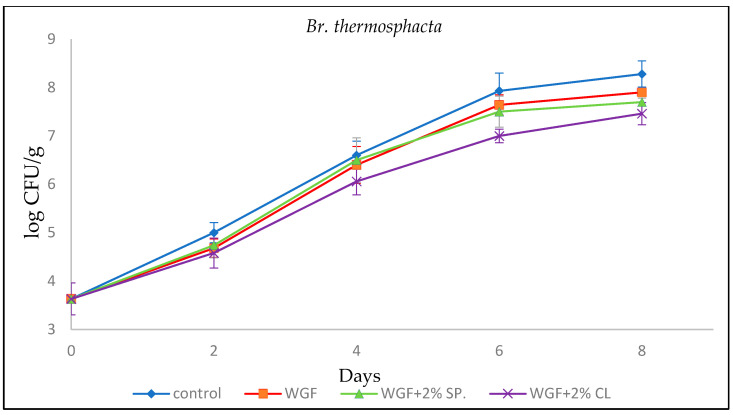
Growth of *Br. Thermosphacta* in minced chicken packaged in different types of films during storage.

*Br. Thermosphacta* is a facultative anaerobic microorganism and represents a significant part of the microbial flora of meat stored both aerobically and in vacuum. The initial population of *Br. Thermosphacta* in minced chicken was 3.63 ± 0.33 log CFU/g (Figure 3), which is consistent with the study conducted by Chouliara et al. [32] who reported an initial population of 3.04 ± 0.21 log CFU/g. On the eighth day of the experiment, the values recorded for this microorganism for Control, WGF, WGF + 2% SPR, and WGF + 2% CL samples were 8.28 ± 0.27, 7.90 ± 0.06, 7.70 ± 0.08, and 7.46 ± 0.23, respectively.

Tsironi et al. [19] studied the effect of whey protein films reinforced with ginger and rosemary essential oils on minced lamb. The initial value of *Br. thermosphacta* was 3.28 log CFU/g and reached its maximum on the 11th day for the whey protein films (Control) (8.01 log CFU/g). A reduction was detected even from the second day while, on the eighth day, it was observed that the addition of essential oils had a positive effect on the inhibition of the growth of *Br. thermosphacta*, as their values remained below 7.15 log CFU/g found in the whey protein film (Control).

#### 3.2.4. *Pseudomonas* spp.

**Figure 4:**
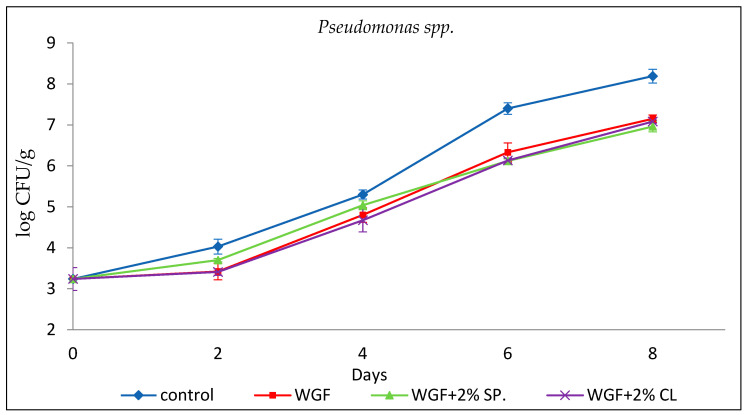
Growth of *Pseudomonas* spp. in minced chicken packaged in different types of films during storage.

*Pseudomonas* spp. is a class of strictly aerobic microorganisms considered spoilage organisms for red meat and poultry. Their growth is associated with the formation of malodorous compounds and the appearance of a slimy layer on the surface of chicken [36]. The initial population of *Pseudomonas* spp. in minced chicken was found to be 3.24 ± 0.28 log CFU/g and showed an increasing trend during the experiment (Figure 4), with the maximum value recorded in the Control sample on the eighth day of storage at 8.19 ± 0.17 log CFU/g. These results agree with Chouliara et al. [32], who recorded a value of 3.38 ± 0.18 log CFU/g on day 0 in chicken, and Raeisi et al. [33] who reported a value of 3.60 log CFU/g as the initial *Pseudomonas* spp. population in chicken.

From the results presented in Figure 4, the limit of 7 log CFU/g for Control exceeded on approximately the fifth day. In contrast, the remaining three types of samples reached or exceeded this limit on the eighth day. Therefore, there is a difference of at least one order of magnitude between the Control and the samples packaged with films. Raeisi et al. [33] reported that the final population of *Pseudomonas* spp. in a chicken sample was 7.8, 7.2, 7.1, and 7 log CFU/g for unpackaged samples, those packaged with sodium alginate film, samples packaged with sodium alginate and nisin film, and samples packaged with sodium alginate and cinnamon EO, respectively. Also, the final population of *Pseudomonas* spp. for the packaged samples with the addition of rosemary EO, cinnamon EO and nisin, rosemary and nisin, and rosemary in combination with cinnamon was 6.5, 6.5, 6.2, and 5.9 log CFU/g, respectively, confirming the antimicrobial activity of EOs and nisin.

#### 3.2.5. *Enterobacteriaceae*

**Figure 5:**
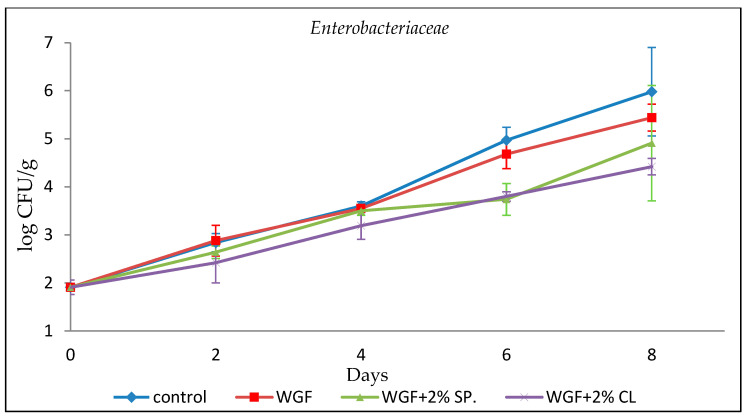
Growth of *Enterobacteriaceae* in minced chicken packaged in different types of films during storage.

Enterobacteria are facultatively anaerobic microorganisms and hygiene indicators in meat production, related to the proper processing and treatment of raw materials. They grow readily in the presence of oxygen; their growth in the absence of oxygen depends on fermentable sugars. Control samples contained more bacteria than WGF-coated samples. From day 6, the number of enterobacteria was greatly smaller in samples that were covered with film containing EOs compared with Control and WGF-coated samples; on day 8 the number of bacteria was up to 1.5 log CFU/g lower than in Control samples. The inhibitory potential of the EO on microbial growth and food spoilage was confirmed. The antimicrobial activity was highest in the WGF + 2% CL film, which maintained the lowest number of enterobacteria during storage (Figure 5). Raeisi et al. [33] reported that under packaging conditions, sodium alginate and eEOs (cinnamon, rosemary) or nisin incorporated into the films reduced *Enterobacteriaceae* by 0.3 to 2 log CFU/g when compared with unwrapped controls, while Bazargani et al. [37] reported that chicken packaged in chitosan antimicrobial films showed a reduced *Enterobacteriaceae* population by 1–2 log CFU/g. Finally, Tsironi et al. [19] recorded that *Enterobacteriaceae* microorganisms of minced lamb (0.45–5.05 log CFU/g) were inhibited by whey films containing 1% ginger and rosemary EOs during their storage period.

#### 3.2.6. Lactic Acid Bacteria (LAB)

**Figure 6:**
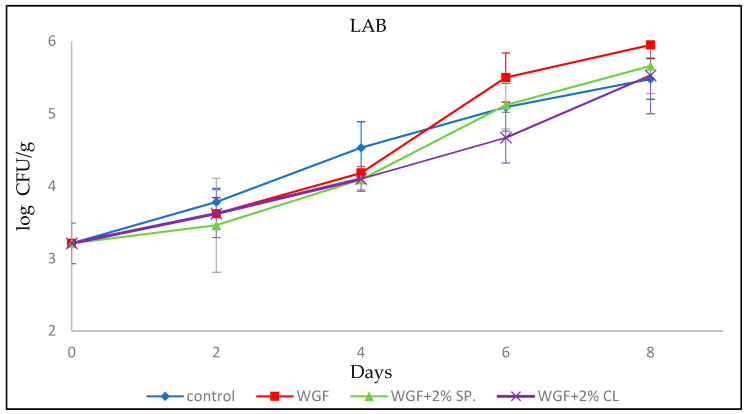
Growth of LAB in minced chicken packaged in different types of films during storage.

The initial LAB population was 3.21 ± 0.28 log CFU/g. Similar values were reported by Chouliara et al. [32] (3.66 ± 0.28 log CFU/g), Raeisi et al. [33] (3.80 log CFU/g), and Gertzou et al. [31] (2.86 log CFU/g). Regarding the present work, the LAB population reached a maximum on the eighth day (5.95 ± 0.18 log CFU/g) for the sample packaged in WGF. Similar values were also observed for the other samples on the same day (Figure 6).

In contrast to the previous microorganisms where the Control samples had the highest bacterial populations throughout the experiment, in the case of LAB this occurred only on the first two days of sampling. In detail, on the second and fourth day, the LAB population in the Control was indeed higher than the other samples, but on the sixth and eighth day of the experiment the sample packaged in WGF showed the highest log CFU/g values. The samples packaged in films enhanced with EOs showed slightly lower values compared with the Control and WGF without however differing significantly from the latter two.

This behavior may be explained to the facultative anaerobic nature of LAB, which allows them to grow efficiently under reduced oxygen availability. Although oxygen permeability was not directly measured in the present study, WG-based films are known to form dense protein networks that can limit oxygen diffusion to some extent. Therefore, the presence of the film in direct contact with the surface of the minced chicken may have contributed to locally reduced oxygen availability compared with the Control samples, favoring LAB growth in the later stages of storage.

The slightly lower LAB populations observed in samples packaged with EO-enhanced WGFs compared with plain WGF may suggest that, despite the potentially favorable oxygen conditions, the antimicrobial activity of spearmint and clove EOs exerted an inhibitory effect on LAB growth. This suggests that the dynamics of LAB in the present study resulted from a combined effect of altered microenvironmental conditions induced by the film matrix and the antimicrobial activity of EOs. However, since oxygen levels were not monitored, this interpretation should be considered suggestive rather than definitive.

Overall, the antimicrobial efficacy followed the trend WGF + 2% CL > WGF + 2% SPR > WGF > Control, highlighting the importance of the EO and its distribution within the film, while the observed results of the present study demonstrate their effectiveness under aerobic refrigeration conditions. Furthermore, unlike previous studies that focused mainly on film characterization [16,25,38], the present study correlates the mechanical, antioxidant, and antimicrobial properties of the film with the actual food preservation results, especially in minced chicken, highlighting how material-level modifications translate into food-level quality preservation. Previous studies involving modified atmosphere packaging (MAP) or antimicrobial coatings have reported significant shelf-life extensions for chicken products. For example, pectin-based (2% *w*/*w* aqueous solution) edible coatings enriched with 1% extract of citrus bioflavonoids (flavomix) or 0.5% glucono-δ-lactone extended the microbial acceptability of chicken thighs to 11–13 days at 5 °C compared with 6–7 days in controls [39]. In addition, vacuum packaging and antimicrobial agents have extended the shelf life of chicken patties from approximately 4 days under aerobic packaging to up to 12 days with combined treatments [40].

The incorporation of EOs can alter film permeability by increasing the heterogeneity of the matrix, leading to changes in oxygen permeability that could indirectly affect microbial growth under aerobic storage conditions. Such conditions, as in the present study, can accelerate microbial growth, while the films can locally reduce oxygen exposure, partially mitigating this phenomenon by inhibiting microbial growth.

Regarding the antimicrobial efficacy of EO-enhanced films, it may be affected by the volatility during drying and storage, as well as by the release kinetics of the bioactive compound on the food surface. Summarizing the results of the microbiological analyses, it is evident that the antimicrobial activity was most pronounced during the first days of storage, suggesting an initial burst of EO components release followed by gradual depletion, a behavior commonly reported for EO-based films. Furthermore, interactions between the bioactive components of EOs and gluten proteins (e.g., hydrophobic interactions or hydrogen bonds) may reduce the bioavailability of the active compounds, which may explain the differences in the antimicrobial performance of clove oil and spearmint EO.

Finally, the antimicrobial activity observed in WGF samples suggests an intrinsic protective effect of the gluten matrix, while the enhanced inhibition in EO-enhanced films demonstrates the additional contribution of bioactive compounds.

### 3.3. Physicochemical Analyses

#### 3.3.1. pH of Packaged Minced Chicken Samples

pH is directly related to the existence and growth of microorganisms, mainly due to the metabolic substances produced during their growth. For example, *Pseudomonas* produce various amino acids which lead to an increase in pH value, in contrast to lactic acid bacteria that produce lactic acid as product which leads to a decrease in pH [36].

The change in pH for the different types of packaged minced chicken samples as a function of storage time is presented in Table 5. The initial pH value of the sample was 6.06 ± 0.03, which is within the range of normal values for fresh, raw meat, while the final value reached 6.85 ± 0.00 for the Control. The increase in the Control sample may be due to the action of endogenous or microbial enzymes such as protease and lipase which cause an increase in volatile bases (e.g., ammonia and trimethylamine) during prolonged storage. Chouliara et al. [32] reported a slightly higher initial pH value (6.40) for chicken meat while Bazargani et al. [37] reported 5.90.

The values for second and fourth day of sampling were similar for all packaging types. From the sixth day onwards, a significant difference was seen between the Control and the different films which continued to have similar values until the end of the experiment. More specifically, on day 6, the Control had a value of 6.56 ± 0.02, while WGF, WGF + 2% SPR, and WGF + 2% CL had values of 6.20 ± 0.14, 6.22 ± 0.17, and 6.19 ± 0.21, respectively. Since pH is associated with the poor condition and spoilage of food, the results from the pH measurements of the samples confirm the microbiological results for preventing spoilage of the packaged food when WGFs are used. This was observed on day 6 and 8 of the experiment where there is inhibition in the growth of microorganisms, maintaining the pH values of the three samples at similar levels at the same time as when the Control increases significantly.

#### 3.3.2. Color of Packaged Minced Meat Samples

Regarding the L* parameter, a general decrease was observed for all samples from day 0 to day 8. As shown in Table 5, the initial L* value was 59.28 ± 6.13, while by the eighth day it had declined to 57.30 ± 2.39 for the Control, 57.21 ± 1.77 for WGF, 57.02 ± 1.36 for WGF + 2% SPR, and 58.17 ± 2.80 for WGF + 2% CL. This reduction indicates a gradual loss in brightness in the minced chicken samples, mainly due to microbial spoilage over time. The increase in bacterial populations, particularly on the surface, has likely caused slime formation and a cloudy appearance, thereby diminishing brightness. Additionally, the decline in L* values may be linked to structural changes in the meat, especially protein denaturation. A slightly higher L* value was recorded for the WGF + 2% CL sample on day 8, suggesting a minor improvement in surface brightness.

For the a* parameter (redness), all samples followed a similar trend. The initial value (10.08 ± 1.12) remained almost stable until day 2, decreased sharply by day 4, and increased again by days 6 and 8, surpassing the initial values for all packaging types. These variations are attributed to myoglobin oxidation and the formation of metmyoglobin during storage. Between days 6 and 8, redness slightly decreased in the Control but increased in the coated samples. On day 8, a* values were 11.15 ± 1.17 for the Control, 12.32 ± 1.29 for WGF, and 12.43 ± 1.71 for WGF + 2% SPR, indicating that the films helped preserve or enhance the red hue preferred by consumers.

Concerning the b* parameter (paleness), values ranged from 17.2 ± 1.26 (day 0) to a maximum of 21.64 ± 0.98 (day 2, WGF + 2% CL). Positive b* values indicate yellowness, while negative values indicate blueness. Although no consistent trend was observed, all samples showed higher final b* values compared with day 0. The Control and WGF + 2% SPR reached peak values on day 8, WGF on day 6, and WGF + 2% CL on day 2. At the end of storage, the Control had the highest b* (20.08 ± 0.42) and WGF + 2% CL the lowest (18.74 ± 0.90).

Overall, the use of WGF did not cause discoloration in minced chicken; instead, in some cases, they enhanced color stability. Thus, all types of films positively influenced the color parameters of the samples.

Gertzou et al. [31] and Chouliara et al. [32] recorded initial L* values of 60.55 and 49.50, respectively, in chicken samples. Kiarsi et al. [41] observed a general decline in L* during storage but noted that beef coated with sage seed films enriched with nutmeg EO maintained the highest brightness. For the a* parameter, Chouliara et al. [32] reported an initial value of 3.30 ± 0.19, whereas Kiarsi et al. [41] found a value of approximately 18.00, with redness decreasing over time for all treatments. Samples with higher EO concentrations showed the lowest redness by the final day. Tsironi et al. [19], studying minced lamb, found that a* slightly decreased during storage except in samples packaged with whey films containing 1% ginger or rosemary EOs, where it increased slightly. Regarding the b* value, Gertzou et al. [31], Chouliara et al. [32], and Kiarsi et al. [41] reported initial values of 9.68, 11.40, and 10.20, respectively—slightly lower than in the present study. Tsironi et al. [19] also observed an increase in b* during storage, similar to the present study, while Kiarsi et al. [41] reported stable or increased b* values for coatings containing 1.5–2% nutmeg EO, likely due to the yellowish color of the coatings themselves.

Although sensory evaluation was not performed, the incorporation of EOs in WGFs may affect not only appearance but also odor perception. The relatively low concentration used (2%) was chosen to primarily balance antimicrobial efficacy with potential sensory impact, which should be further evaluated in future studies.

### 3.4. Composition of Essential Oils

Table 6 shows the composition of the EOs used in the present study. Thirteen volatile compounds were identified and semi-quantified in spearmint EO, while only five compounds were found in clove EO. The main compounds of spearmint EO were D-(+)-carvone (77.72 ± 4.33%), followed by dl-limonene (9.97 ± 1.15%) and menthol (3.64 ± 0.96%). On the other hand, clove EO was mainly composed of eugenol (75.62 ± 3.56%), eugenyl acetate (16.10 ± 2.54%), and caryophyllene (6.58 ± 1.15%). In both cases, other compounds were present at lower concentrations. The compounds identified have been reported to possess significant antimicrobial activity with clove oil having a significantly greater effect, in comparison to spearmint, as its largest proportion consists of eugenol, with strong antimicrobial activity, while spearmint oil has a higher percentage of D-(+)-carvone [12]. This is probably the reason why the film samples with clove EO appeared to be the most active against the growth of microorganisms.

As EOs are inherently volatile compounds, some losses may be observed during the drying and storage of the film, especially at high temperatures. This may explain the reduced antimicrobial effect observed at later stages of storage. The antimicrobial efficacy of active films depends on the migration of EO components to the food surface and not only on the initial loading. The gradual release from the protein matrix may explain the stronger antimicrobial effects observed during the early storage period. Although the retention and release kinetics of EOs were not directly measured in the present study, the sustained antimicrobial activity recorded throughout the storage period suggests that a fraction of bioactive compounds remained available at the food–film interface. Encapsulation strategies, which have been shown to improve EO stability and control release [42], were not applied in the present study, as the primary objective was to evaluate the performance of wheat gluten films incorporating free essential oils in a simple formulation. This approach was chosen to maintain process simplicity and potential industrial feasibility. Nevertheless, the encapsulation of essential oils within protein-based matrices represents an important direction for future research.

## 4. Conclusions

The WGFs, plain and enhanced with spearmint and clove essential oils, exhibited enhanced antimicrobial and antioxidant activity that affected the microbiological quality of minced chicken under aerobic, refrigerated storage (4 ± 1 °C). WGF + 2% CL showed the strongest antimicrobial performance, extending the shelf life of minced chicken by 2 days compared with the Control. The incorporation of essential oils contributed to the delay of microbial growth, while LAB growth was not inhibited, probably due to the combined effect of storage conditions and packaging environment. Furthermore, improvements in pH stability and color maintenance supported the positive effect of the films on product quality during 8 days of storage. The incorporation of essential oils increased the flexibility and elasticity of wheat gluten films, while the tensile strength decreased, indicating a plasticizing effect on the protein matrix, with clove essential oil being the most effective. FTIR analysis showed that the essential oils were physically entrapped within the film structure rather than chemically bound.

Overall, the results indicate that wheat gluten-based films incorporating essential oils influenced food preservation, confirming the potential of active biodegradable packaging for minced poultry under the specific experimental conditions evaluated. However, the conclusions are limited to the studied storage conditions, essential oil concentrations, and the absence of sensory evaluation, permeability measurements, and release kinetics. Further research is needed to evaluate sensory acceptability, essential oil migration behavior, long-term antimicrobial performance, and comparison with commercial packaging systems before broader industrial application is considered. Environmental benefits associated with biodegradability are intrinsic properties of the materials but were not experimentally evaluated in this study.

## Figures and Tables

**Table 1:** Mean values and standard deviation (μm) of thickness for the 3 different types of prepared films.

Film Type	Thickness (μm)
WGF	176.0 ± 50.5 ^A^
WGF + 2% SPR	145.2 ± 19.0 ^A^
WGF + 2% CL	281.5 ± 37.0 ^B^

^A,B^ Mean values with different superscripts indicate statistically significant differences between film types (*p* < 0.05).

**Table 2:** Mean values and standard deviation of L*, a*, and b* parameters for the three different types of prepared films.

Film Type	L*	a*	b*
WGF	71.90 ± 0.51 ^B^	−3.26 ± 0.01 ^A^	13.13 ± 0.86 ^A^
WGF + 2% SPR	71.73 ± 0.03 ^B^	−2.92 ± 0.04 ^B^	13.87 ± 0.23 ^A^
WGF + 2% CL	69.18 ± 1.29 ^A^	−1.03 ± 0.90 ^C^	21.29 ± 1.57 ^B^

^A,B,C^: Mean values in each column with different superscripts indicate statistically significant differences between film types (*p* < 0.05).

**Table 3:** Mean values and standard deviation of % percent of antioxidant activity for the three different types of prepared films.

Film Type	% A.A.
WGF	29.18 ± 3.03 ^A^
WGF + 2% SPR	50.22 ± 11.47 ^B^
WGF + 2% CL	88.76 ± 1.19 ^C^

^A,B,C^: Mean values with different superscripts indicate statistically significant differences between film types (*p* < 0.05).

**Table 4:** Mean values and standard deviation of mechanical property values for the three different types of prepared films.

Film Type	Elongation at Break (%)	Tensile Strength at Break (MPa)	Young’s Modulus (MPa)
WGF	244.2 ± 21.4 ^A^	432.0 ± 47.6 ^B^	4107.5 ± 205.8 ^A^
WGF + 2% SPR	242.1 ± 30.6 ^A^	300.6 ± 14.1 ^A^	2786.7 ± 187.0 ^A^
WGF + 2% CL	272.4 ± 24.8 ^A^	356.5 ± 87.1 ^A^	3165.5 ± 37.5 ^A^

^A,B^: Mean values in each column with different superscripts indicate statistically significant differences between film types (*p* < 0.05).

**Table 5:** Change in pH value and color parameters during storage of minced chicken samples packaged with different types of films.

Parameter	Day	Control	WGF	WGF + 2% SPR	WGF + 2% CL
pH	0	6.06 ± 0.03 ^A^	6.06 ± 0.03 ^A^	6.06 ± 0.03 ^A^	6.06 ± 0.03 ^A^
	2	6.17 ± 0.17 ^a,A^	6.17 ± 0.18 ^a,A^	6.16 ± 0.28 ^a,A^	6.11 ± 0.23 ^a,A^
	4	6.15 ± 0.06 ^a,A^	6.13 ± 0.13 ^a,A^	6.14 ± 0.23 ^a,A^	6.15 ± 0.22 ^a,A^
	6	6.56 ± 0.02 ^a,B^	6.20 ± 0.14 ^a,A^	6.22 ± 0.17 ^a,A^	6.19 ± 0.21 ^a,A^
	8	6.85 ± 0.00 ^a,C^	6.28 ± 0.10 ^b,A^	6.23 ± 0.10 ^b,A^	6.28 ± 0.25 ^b,B^
L*	0	59.28 ± 6.13 ^a,A^	59.28 ± 6.13 ^a,A^	59.28 ± 6.13 ^a,A^	59.28 ± 6.13 ^a,A^
	2	59.64 ± 3.40 ^a,A^	57.35 ± 1.44 ^a,A^	57.82 ± 0.57 ^a,A^	58.47 ± 1.85 ^a,A^
	4	58.44 ± 6.37 ^a,A^	57.09 ± 5.05 ^a,A^	56.36 ± 6.37 ^a,A^	56.88 ± 5.87 ^a,A^
	6	57.64 ± 2.06 ^a,A^	58.14 ± 2.93 ^a,A^	56.74 ± 1.11 ^a,A^	58.02 ± 2.00 ^a,A^
	8	57.30 ± 2.39 ^a,A^	57.21 ± 1.77 ^a,A^	57.03 ± 1.36 ^a,A^	58.17 ± 2.80 ^a,A^
a*	0	10.08 ± 1.12 ^a,A^	10.08 ± 1.12 ^a,A^	10.08 ± 1.12 ^a,A,B^	10.08 ± 1.12 ^a,A^
	2	10.03 ± 1.37 ^a,A^	10.72 ± 1.28 ^a,A^	10.62 ± 3.00 ^a,A,B^	10.75 ± 0.51 ^a,A^
	4	7.20 ± 0.12 ^a,A^	6.91 ± 0.07 ^a,A^	7.49 ± 0.56 ^a,A^	6.78 ± 0.34 ^ba,A^
	6	11.34 ± 1.30 ^a.A^	12.00 ± 1.62 ^a,A^	12.17 ± 1.51 ^a,A,B^	11.08 ± 1.20 ^a,A^
	8	11.15 ± 1.17 ^a,A^	12.32 ± 1.29 ^a,A^	12.43 ± 1.71 ^a,B^	11.68 ± 0.80 ^a,A^
b*	0	17.21 ± 1.26 ^a,A^	17.2 ± 1.26 ^a,A^	17.21 ± 1.26 ^a,A^	17.21 ± 1.20 ^a,A^
	2	19.60 ± 2.51 ^a,A^	19.64 ± 1.44 ^a,A^	19.76 ± 1.25 ^a,A^	21.64 ± 0.90 ^a,A^
	4	17.41 ± 1.27 ^a,A^	18.78 ± 2.88 ^a,A^	18.80 ± 1.34 ^a,A^	19.42 ± 1.60 ^a,A^
	6	18.95 ± 1.20 ^a,A^	20.05 ± 0.72 ^a,A^	19.46 ± 0.88 ^a,A^	20.58 ± 0.20 ^a,A^
	8	20.08 ± 0.42 ^a,A^	19.78 ± 0.43 ^a,A^	19.80 ± 1.08 ^a,A^	18.74 ± 0.90 ^a,A^

^a,b^: Different superscripts in each row indicate statistically significant differences (*p* < 0.05) for a specific day of storage for packaging with each film type. ^A,B,C^: Different superscripts in each column indicate statistically significant differences (*p* < 0.05) between days for packaging with a film type.

**Table 6:** Composition (mean value ± standard deviation) of clove and spearmint EOs.

Library/ID	% Composition
Spearmint essential oil	
(-)-α-Pinene	0.60 ± 0.04
β-Pinene	0.73 ± 0.09
dl-Limonene	9.97 ± 1.15
Eucalyptol	0.68 ± 0.01
Menthone	1.48 ± 0.55
Menthol	3.64 ± 0.96
4-Terpineol	1.58 ± 0.14
α-Terpineol	0.71 ± 0.12
Dihydrocarbone	0.63 ± 0.11
D-(+)-Carvone	77.72 ± 4.33
Piperitone	0.32 ± 0.14
Caryophyllene	1.20 ± 0.14
(-)-Caryophyllene Oxide	0.74 ± 0.222
Clove essential oil	
Eugenol	75.62 ± 3.56
Caryophyllene	6.58 ± 1.15
α-Caryophyllene	0.92 ± 0.04
Eugenyl acetate	16.10 ± 2.54
(-)-Caryophyllene oxide	0.78 ± 0.14

## Data Availability

The original contributions presented in this study are included in the article/Supplementary Materials. Further inquiries can be directed to the corresponding authors.

## Supplementary Materials

The following supporting information can be downloaded at: https://www.mdpi.com/article/10.3390/foods15020390/s1, Figure S1: Films placed on filter paper (a) WGF, (b) WGF + 2% SPR, (c) WGF + 2% CL; Figure S2: Minced chicken packed in wheat gluten films placed on a polystyrene tray; Figure S3: Minced chicken packed in wheat gluten films placed on a polystyrene tray and wrapped with low-density polyethylene film; Figure S4: FTIR-ATR spectra of WGF (blue line), WGF + 2% SPR (yellow line) and WGF + 2% CL (orange line) films.
