# Supplementary material for: Effect of Wheat Gluten Films Infused with Mint and Clove Essential Oils on the Shelf Life of Fresh Minced Chicken
Source: Foods. 2026 Jan 21;15(2):390. doi: 10.3390/foods15020390 (PMC12840709; doi:10.3390/foods15020390)
Supplement: Supplementary file 1 [file foods-15-00390-s001.zip › foods-4067561-supplementary.pdf]

foods

Supplementary File

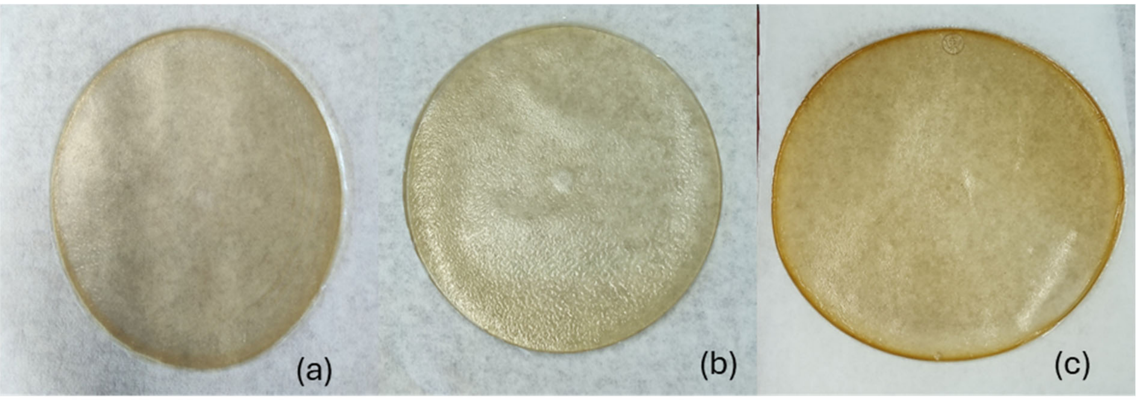

Figure S1. Films placed on filter paper (a) WGF, (b) WGF+2%SPR, (c) WGF+2%CL.

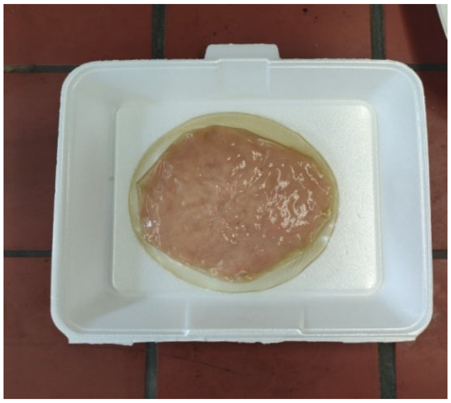

Figure S2. Minced chicken packed in wheat gluten films placed on a polystyrene (PS) tray.

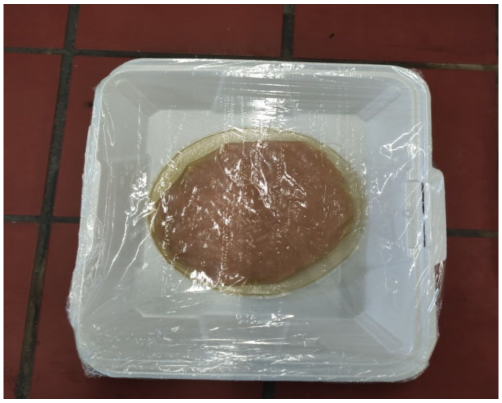

Figure S3. Minced chicken packed in wheat gluten films placed on a polystyrene (PS) tray and wrapped with low-density polyethylene (LDPE) film.

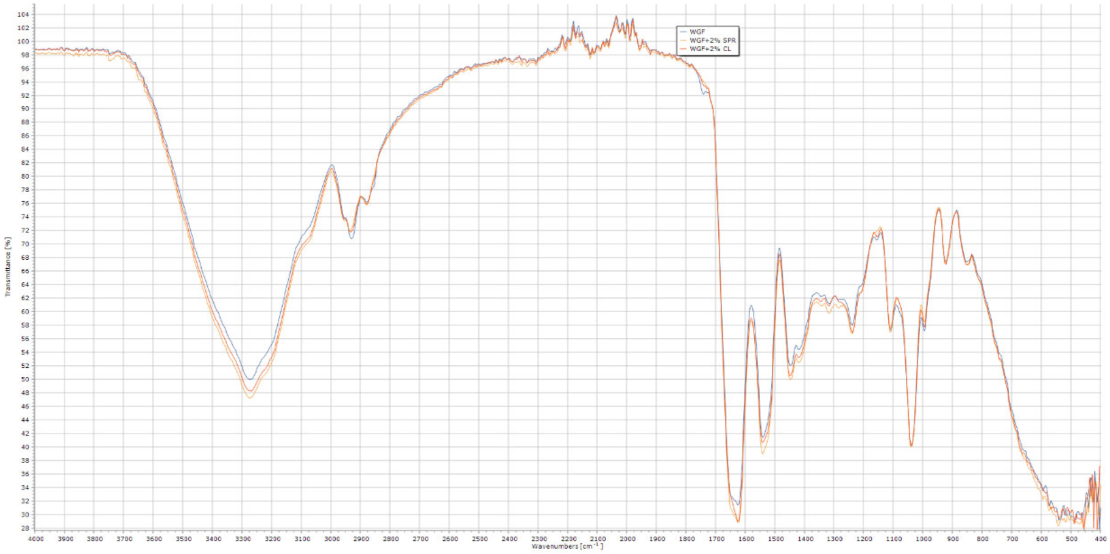

Figure S4. FTIR-ATR spectra of WGF (blue line), WGF+2% SPR (yellow line) and WGF+2% CL (orange line) membranes.
